# Evolution of out-of-home food consumption in Brazil in 2008–2009 and 2017–2018

**DOI:** 10.11606/s1518-8787.2021055003221

**Published:** 2021-11-12

**Authors:** Ilana Nogueira Bezerra, Thaís M Vasconcelos, Jessica Brito Cavalcante, Edna Massae Yokoo, Rosângela A Pereira, Rosely Sichieri

**Affiliations:** I Universidade Estadual do Ceará Centro de Ciências da Saúde Programa de Pós-Graduação em Nutrição e Saúde Fortaleza CE Brasil Universidade Estadual do Ceará. Centro de Ciências da Saúde. Programa de Pós-Graduação em Nutrição e Saúde e Programa de Pós-Graduação em Saúde Coletiva. Fortaleza, CE, Brasil; II Universidade Estadual do Ceará Programa de Pós-Graduação em Saúde Coletiva Fortaleza CE Brasil Universidade Estadual do Ceará. Programa de Pós-Graduação em Saúde Coletiva. Fortaleza, CE, Brasil; III Universidade Estadual do Ceará Programa de Pós-Graduação em Nutrição e Saúde Fortaleza CE Brasil Universidade Estadual do Ceará. Programa de Pós-Graduação em Nutrição e Saúde. Fortaleza, CE, Brasil; IV Universidade Federal Fluminense Departamento de Epidemiologia e Bioestatística Niterói RJ Brasil Universidade Federal Fluminense. Departamento de Epidemiologia e Bioestatística. Niterói, RJ, Brasil; V Universidade Federal do Rio de Janeiro Departamento de Nutrição Social e Aplicada Rio de Janeiro RJ Brasil Universidade Federal do Rio de Janeiro. Departamento de Nutrição Social e Aplicada. Rio de Janeiro, RJ, Brasil; VI Universidade do Estado do Rio de Janeiro Instituto de Medicina Social Departamento de Epidemiologia Rio de Janeiro RJ Brasil Universidade do Estado do Rio de Janeiro. Instituto de Medicina Social. Departamento de Epidemiologia. Rio de Janeiro, RJ, Brasil

**Keywords:** Feeding Behavior, Food Services, Collective feeding, Restaurants, Street Food, Diet Surveys

## Abstract

**OBJECTIVE:**

To describe the evolution of out-of-home food consumption in Brazil in 2008–2018.

**METHODS:**

We used the 2008–2009 and 2017–2018 data from the *Inquéritos Nacionais de Alimentação* (INA - National Food Surveys), conducted amid 34,003 and 46,164 individuals, to estimate the frequency of out-of-home food consumption and the contribution of this consumption to specific foods. Food consumption was analyzed using food records in the 2008–2009 INA and 24-hour recalls in 2017–2018. Estimates were generated for Brazil in general, for urban and rural areas, for age groups (adolescent, adult, elderly), and for income bracket.

**RESULTS:**

The frequency of out-of-home consumption decreased by 8.8% between the two surveys, with no change in the rural area, in the Northeast and South regions, and for the lowest income brackets. We observed a slight increase among the elderly and in the Midwest region. The contribution of out-of-home food consumption to daily energy intake also decreased (16.3% *vs.* 12.7%), excepting the rural area, where there was a reduction in the difference in relation to the urban area between the two surveys. For most items evaluated, the out-of-home food consumption decreased. The most consumed out-of-home food were alcoholic beverages, fried and baked snacks, soft drinks, pizza, sweets, and sandwiches in both surveys.

**CONCLUSION:**

In 10 years, the prevalence of food consumption and the percentage of contribution of out-of-home food decreased in Brazil, but ultra-processed foods still figure as the most consumed food group outside the home.

## INTRODUCTION

In Brazil, few studies have evaluated food consumption outside home^1^. Some international studies^4^ show that out-of-home consumption is associated with lower nutritional quality and higher intake of energy, carbohydrates, proteins, fats, and sodium. The association between the habit of eating food prepared outside the home and the worst quality of the diet calls attention because this consumption has increased in past years^7^.

Using household food availability data from the *Pesquisas de Orçamentos Familiares* (POF - Household Budget Surveys), Claro et al.^8^ described the evolution of spending on food away from home in Brazil in 2002–2003 and 2008–2009. Researchers found an increasing trend in this habit, more accentuated at higher income levels, but present in all regions of the country and in urban and rural areas^8^. However, the evolution of what is effectively consumed away from home has not yet been analyzed. This article describes for the first time the evolution of out-of-home food consumption during 10 years, based on data from the *Inquéritos Nacionais de Alimentação* (INA - National Food Surveys) conducted together with the POF in 2008–2009 and 2017–2018.

## METHODS

We analyzed data from the INA and the POF’s food consumption module, both conducted by the Brazilian Institute of Geography and Statistics (IBGE) in 2008–2009 and 2017–2018.

Based on the *Sistema Integrado de Pesquisas Domiciliares* (Integrated Household Survey System), the two surveys adopted a two-stage cluster sampling plan, corresponding to a “master sample”, common to all IBGE household surveys. The master sample comprises census tracts, which are the primary sampling units.

The sectors went through a stratification scheme that allows the generation of estimates for the five Brazilian regions, for the rural and urban areas, and for different socioeconomic levels. The sectors were selected by sampling with probability proportional to the number of households in the sector, within each final stratum. The subsamples of primary units for the POF were selected by simple random sampling in each stratum. Secondary units were permanent private households, selected by simple random sampling from each of the selected and stratified primary units. Twenty-five percent of POF households were selected to participate in the INA. In POF 2008–2009, 13,569 households participated in the INA; in POF 2017–2018, it was 20,112.

The INA included all residents over 10 years of age in the selected households, totaling 34,003 individuals in 2008–2009 and 46,164 individuals in 2017–2018. A detailed description of the sampling of the two surveys is available in the official IBGE publications^9,10^. Data collection from the surveys was carried out over 12 months, uniformly across the strata, ensuring representativeness in the four quarters of the year.

In the 2008–2009 survey, data were collected by applying two food records with information on food and beverages consumed, type of preparation, quantity, time, and place of consumption (at home or away from home ) on non-consecutive days. In 2017–2018, the collection took place during 24-hour recalls on two non-consecutive days. Individuals were interviewed in person by a trained research agent, who interrogated him and recorded all food and drink consumed the day before each visit. The interview script, based on the multiple-pass method, was structured in sequential stages^11^. IBGE processed the data in a specific program.

In the 2017–2018 edition, besides items already investigated in the previous edition, researchers included some “addition items”, with products usually added to foods such as bread, pasta, beverages, etc.: butter/margarine, sauces, and sugar or sweetener. For the present study, considering that the 2008–2009 consumption module did not cover some information and that data analysis in the 2017–2018 edition would generate mistakenly high estimates, the addition items were excluded to estimate percentages of foods consumed outside the home, taking into account only spontaneous records, without survey questions.

In both surveys, a trained research agent analyzed the reports together with the respondent at the end of the visit, looking for possible filling mistakes and omissions of commonly forgotten foods (candies, small snacks, etc.). In case of absence or doubt in the record of the unit of measurement used, the respondents were asked to present the measurement tool to the agent, so that he could insert into the system the corresponding household measurement.

Out-of-home food consumption included all foods and beverages purchased outside the home and consumed without going through the household supply. In 2017–2018, this information was collected in more detail, with options to specify the place of consumption. For the present study, we evaluated the first day of food consumption, considering out-of-home food consumers those individuals who reported consuming at least one item away from home.

Questionnaires with socioeconomic and demographic data, containing information on the age and gender of the residents and *per capita* family income, were answered by the reference person in the household. Per capita family income was stratified based on the minimum wage in force at the time of the surveys (R$415.00 in 2008–2009 and R$954.00 in 2017–2018): up to 0.5 minimum wage, 0, 5 to 1 minimum wage, 1 to 2 minimum wages, and 2 or more minimum wages. Age was assessed based on three age groups: adolescents (10 to 19 years old), adults (20 to 59 years old), and elderly people (60 years old or older).

The foods mentioned in the two surveys were categorized according to the nutritional and consumption characteristics of the items into 26 groups: rice; beans and other legumes; green leafy vegetables; other vegetables; tuberoses; fruits; pastas; baked goods; sweet cakes and cookies; industrialized snacks and salted cookies; beef; swine; poultry; fish and seafood; processed meats; eggs; milk and dairy products; candy; sauces and oils; alcoholic beverages; refreshments and juices; soft drinks; coffee and teas; pizzas; fried and baked snacks; and sandwiches.

The amount of food consumed in grams or milliliters was estimated using the tables of measures referred to for foods consumed in Brazil, referring to each survey^10,12^. Then, the nutritional composition was estimated from the same nutritional composition table generated for the 2017–2018^10^ survey.

The contribution of eating away from home to the total consumption of each food group and to the total energy intake (proportion consumed outside the home) was estimated using the ratio of means method, according to sociodemographic and economic characteristics (age groups, gender, Brazilian regions, urban and rural areas, and socioeconomic strata). We generated estimations separately for each survey and their 95% confidence intervals were compared to identify changes over time.

The time of consumption of food was evaluated, considering five different periods: from 7 am to 10 am, from 11 am to 2 pm, from 3 pm to 6 pm, from 7 pm to 10 pm, from 11 pm to 6 am. Intermediate times that were recorded within 29 minutes of the hour were recorded in the previous hour and times above 30 minutes were recorded in the later hour. For example, consumptions made between 2:01 pm and 2:29 pm were registered as 2:00 pm, and consumption between 2:30 pm and 2:59 pm as 3:00 pm.

For the 2017–2018 INA, we assessed specific consumption locations, estimating their contribution to energy intake away from home. The following locations were considered: school (consumption reported in a school environment, including universities), restaurants (*à la carte* and *pay per kilo*), bar and street (bars, snack bars, fast food, street food), and other places (consumption away from home in places not classified in the previous groups, such as gas stations, pharmacy, supermarkets, etc.).

Analyzes were performed with the SAS software version 9.4, considering the sample weight and using the *survey* procedure to incorporate the complexity of the sample.

## RESULTS

**Figure 1:**
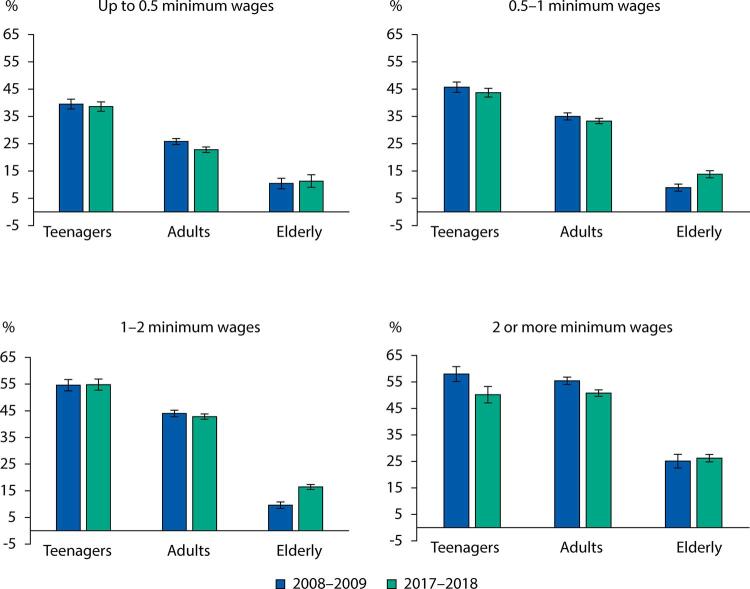
Frequency of individuals who consume food away from home, according to income and age. Brazil, 2008–2009 and 2017–2018.

Between 2008 and 2018 there was a reduction in the frequency of out-of-home food consumption in Brazil, in the North and Southeast regions, in the urban area, among men and women, adolescents and adults, and among individuals with higher incomes. No changes were observed in the Northeast and South regions, in the rural area, among the elderly, and in the lowest income brackets. The Midwest region was the only one that showed an increase in the frequency of consumption away from home (Table 1). The increase in out-of-home food consumption was also observed among elderly people with *per capita* household income between 1 and 2 minimum wages (Figure 1).

**Table 1:** Frequency (and 95% confidence interval) of individuals who consume food away from home, according to sociodemographic variables. Brazil, 2008– 2009 and 2017–2018.

Variables	2008–2009 (n = 34,003)	2017–2018 (n = 46,164)
	
	% (95%CI)	% (95%CI)
BRAZIL Total	40.2 (39.1–41.3)	36.5 (35.6–37.5)
**Domicile situation**		
Urban	42.8 (41.5–44.0)	38.0 (36.9–39.1)
Rural	27.3 (25.4–29.3)	27.6 (26.0–29.3)
**Region**		
North	42.6 (40.2– 45.0)	30.5(27.8–33.1)
Northeast	33.5 (31.9–35.1)	34.8 (33.5–36.2)
Southeast	43.7 (41.5–45.8)	36.1 (34.3–37.9)
South	40.1 (38.1–43.2)	38.5 (36.3–40.5)
Midwest	41.9 (39.2–44.7)	47.7 (45.0–50.4)
**Sex**		
Man	44.4 (43.0–45.9)	40.0 (38.8–41.2)
Woman	36.3 (35.0–37.6)	33.3 (32.2–34.4)
**Age**		
Adolescents	48.1 (45.9–50.2)	43.4 (41.4–45.4)
Adults	42.6 (41.3–43.9)	39.3 (38.2–40.4)
Older adults	16.1 (13.6–18.5)	19.4 (18.0– 20.8)
**Per capita household income**		
Up to 0.5 minimum wage	30.1 (28.0–32.2)	27.1 (25.3–29.0)
1 minimum wage	35.3 (33.2–37.5)	33.3 (31.7–34.9)
1 to 2 minimum wages	40.2 (38.2–42.3)	37.3 (35.6–39.0)
≥ 2 minimum wages	50.1 (47.7–52.4)	44.6 (43.6–46.6)

Similar results related to frequency were observed in the energy contribution of eating away from home to total energy intake, with a reduction of 3.6 percentage points (pp). The greatest reduction was observed in the North region (5.6 pp) and the percentages did not change for the Northeast and Center-West regions. The participation of eating away from home also did not change for individuals living in rural areas and for older adults (Table 2).

**Table 2:** Evolution of the energy contribution of eating away from home according to sociodemographic variables. Brazil, 2008–2009 and 2017–2018.

Variables	2008–2009	2017–2018
	
	% (95%CI)	% (95%CI)
BRAZIL Total	16.3 (15.7–17.0)	12.7 (12.2–13.3)
**Area**		
Urban	17.6 (16.9–18.3)	13.4 (12.8–14.0)
Rural	10.0 (9.1–11.0)	9.0 (8.1–9.8)
**Regions**		
North	16.2 (14.8–17.6)	10.6 (9.5–11.7)
Northeast	13.5 (12.6–14.3)	12.4 (11.6–13.1)
Southeast	17.7 (16.5–18.9)	12.4 (11.4–13.3)
South	16.9 (15.5–18.5)	13.9 (12.5–15.3)
Midwest	17.7 (15.6–19.8)	16.0 (14.6–17.4)
**Sex**		
Man	17.8 (17.0–18.6)	13.6 (13.0–14.2)
Woman	14.6 (13.9–15.4)	11.7 (11.1–12.3)
**Age**		
Adolescents	15.2 (14.2–16.2)	12.2 (11.4–13.1)
Adults	18.5 (17.7–19.3)	14.6 (13.9–15.2)
Older adults	6.5 (5.2–7.8)	5.6 (4.9–6.1)
**Per capita household income**		
Up to 0.5 minimum wage	11.4 (10.4–12.5)	8.2 (7.5–8.9)
1 minimum wage	13.2 (12.0–14.3)	10.3 (9.6–11.1)
1 to 2 minimum wages	15.8 (14.7–17.0)	12.5 (11.7–13.4)
≥ 2 minimum wages	21.7 (20.3–23.0)	17.8 (16.7–19.1)

Out-of-home alcohol consumption accounted for almost 50% of all alcoholic beverages consumed in 2017–2018. Despite the reduction of out-of-home food consumption by more than 10 percentage points among the surveys, this item continued the one that presents the greatest contribution to out-of-home food consumption. Fried and baked snacks are the second-largest contributors, followed by soft drinks, pizzas, sweets, and sandwiches in 2017–2018; pizzas, sandwiches, soft drinks, and sweets were the second-largest contributors in 2008–2009.

The evolution of consumption of specific foods shows a reduction for most of the items evaluated, but different profiles can be observed according to the household situation. The contribution of out-of-home food consumption in the urban area was greater than in the rural area for almost all groups, excepting for sauces and oils. And the participation of out-of-home consumption of most groups in the rural area showed little or no change when comparing the two surveys. The contribution decreased for fruits, sweets, and sandwiches both for urban and rural areas. In general, we observed a significant reduction in out-of-home consumption of sandwiches (23 pp), pizza (17.8 pp), and fried and baked snacks (13.1 pp). The reduction of sandwich consumption in the urban area was greater than in the rural area 23.4 pp *vs*. 16.1 pp) (Table 3).

**Table 3:** Evolution of the contribution of food groups consumed away from home in Brazil and in urban and rural areas. Brazil, 2008–2009 and 2017–2018.

Food	Brazil		Urban		Rural	
		
	2008–2009 % (95%CI)	2017–2018 % (95%CI)	2008–2009 % (95%CI)	2017–2018 % (95%CI)	2008–2009 % (95%CI)	2017–2018 % (95%CI)
Rice	12.8 (12.1–13.5)	10.4 (9.8–10.9)	14.1 (13.2–14.9)	11.0 (10.3–11.6)	7.2 (6.2–8.2)	7.5 (6.4–8.7)
Beans and other legumes	12.2 (11.4–12.9)	9.4 (8.8–10.0)	13.6 (12.7–14.5)	10.0 (9.3–10.6)	6.8 (5.4–7.7)	6.7 (5.6–7.8)
Green leafy vegetables	18.9 (17.1–20.6)	15.1 (13.9–16.3)	20.2 (18.2–22.2)	15.7 (14.4–17.0)	8.9 (6.6–11.1)	9.6 (7.0–12.2)
Other vegetables	13.1 (10.8–15.4)	12.9 (11.5–14.4)	14.3 (11.8–16.9)	13.9 (12.3–15.6)	7.3 (2.7–11.9)	7.0 (4.4–9.6)
tuberoses	16.3 (14.0–18.6)	14.5 (12.7–16.2)	18.0 (15.4–20.7)	15.5 (13.5–17.6)	8.1 (5.3–10.9)	9.0 (6.5–11.5)
Fruits	15.7 (14.4–17.0)	9.5 (8.6–10.4)	16.4 (14.9–17.9)	10.1 (9.1–11.1)	12.8 (10.5–15.2)	6.2 (4.8–7.7)
Pastas	14.5 (12.7–16.2)	13.0 (11.5–14.4)	15.2 (13.0–17.2)	13.2 (11.5–14.8)	9.8 (7.3–12.3)	11.5 (8.7–14.3)
Baked goods	9.3 (8.6–10.0)	8.1 (7.4–8.7)	9.4 (8.6–10.2)	8.2 (7.5–9.0)	8.6 (6.8–10.3)	6.5 (5.2–7.7)
Sweet cakes and cookies	19.2 (17.2–21.3)	16.0 (14.4–17.5)	20.4 (17.9–22.8)	16.7 (15.0–18.4)	13.6 (10.4–16.9)	11.8 (9.5–14.0)
Industrialized snacks & savory cookies	20.0 (16.5–23.5)	12.9 (10.9–14.4)	21.2 (16.9–25.4)	13.7 (11.3–16.1)	14.8 (10.6–19.0)	9.1 (6.8–11.5)
Bovine meat	16.2 (14.9–17.6)	13.5 (12.5–14.5)	17.8 (16.2–19.4)	14.2 (13.1–15.4)	8.5 (6.7–10.2)	9.2 (7.5–10.9)
Pork	17.0 (12.3–21.8)	10.7 (8.5–13.0)	20.3 (14.0–26.6)	12.5 (9.5–15.4)	9.2 (4.6–13.8)	6.1 (3.7–8.4)
Poultry	17.2 (15.6–18.7)	11.2 (10.3–12.1)	18.5 (16.6–20.3)	11.8 (10.8–12.8)	9.8 (7.5–12.1)	7.9 (6.3–9.5)
Fish and seafood	10.7 (8.7–12.8)	14.1 (11.4–16.8)	12.8 (10.3–15.4)	17.7 (14.0–21.3)	7.0 (3.6–10.4)	5.2 (3.9–7.0)
Processed meats	11.7 (8.9–14.6)	9.6 (7.9–11.2)	11.8 (8.7–15.0)	10.0 (8.1–11.9)	10.8 (7.2–14.4)	6.7 (4.2–9.1)
Eggs	6.7 (5.5–7.4)	6.4 (5.2–7.5)	7.5 (6.1–8.9)	6.5 (5.3–7.8)	3.8 (2.4–5.2)	5.5 (2.3–8.8)
Milk and milk products	7.9 (6.9–8.8)	6.5 (5.6–7.4)	7.9 (6.9–8.9)	8.4 (5.4–7.3)	7.5 (5.1–10.0)	6.9 (5.0–8.8)
Sweets	33.2 (30.2–36.2)	23.3 (20.7–25.9)	33.7 (30.4–37.0)	24.8 (21.9–27.8)	29.8 (23.0–36.5)	15.1 (11.6–18.7)
sauces and oils	8.5 (7.3–9.8)	5.7 (3.6–7.9)	8.8 (7.5–10.2)	5.5 (3.2–7.7)	6.3 (4.1–8.6)	7.2 (0.4–14.0)
Alcoholic drinks	60.8 (53.9–67.7)	49.6 (43.7–55.4)	61.0 (53.5–68.5)	48.6 (42.4–54.8)	59.0 (44.3–73.7)	61.9 (51.4–72.4)
Refreshments and juices	18.5 (17.3–19.8)	14.9 (13.9–15.9)	19.3 (17.9–20.7)	15.6 (14.4–16.7)	13.3 (10.9–15.7)	10.8 (9.3–12.2)
Soft Drinks	40.0 (37.4–42.4)	30.9 (28.5–33.3)	40.2 (37.5–42.9)	30.9 (28.3–33.5)	36.4 (29.5–43.3)	30.8 (25.3–36.4)
coffee and teas	9.9 (9.3–10.6)	9.9 (9.2–10.7)	11.0 (10.2–11.8)	10.8 (10.0–11.7)	5.5 (4.5–6.4)	5.9 (5.0–6.9)
Pizzas	42.5 (31.9–53.1)	24.7 (18.4–31.0)	42.0 (30.7–53.3)	24.6 (18.1–31.1)	52.8 (26.5–79.2)	27.8 (10.9–44.7)
Fried and baked snacks	48.8 (44.3–53.3)	35.7 (32.0–39.4)	51.1 (46.3–56.1)	37.0 (33.1–40.9)	29.1 (20.3–37.8)	25.3 (17.0–33.6)
Sandwiches	41.3 (36.9–45.8)	18.3 (16.4–20.1)	41.8 (37.0–46.6)	18.4 (16.4–20.4)	32.6 (22.0–43.2)	16.5 (11.6–21.4)

In 2008–2009, the time range between 11 am and 2 pm (45.4%) showed the highest frequency of out-of-home food consumption. Another 19.8% were consumed between 7 am and 10 am; 20.1% between 3 pm and 6 pm; 12.2% between 7 pm and 10 pm; and 2.5% between 11 pm and 6 am. In 2017–2018, the frequencies remained in the same order, with 39.9% of foods consumed between 11 am and 2 pm; 19.7% between 7 am and 10 am; 21% between 3 pm and 6 pm; 13.8% between 7 pm and 10 pm and 5.5% between 11 pm and 6 pm.

In 2017–2018, other unspecified places were had the highest percentage of out-of-home food consumption, followed by restaurants, bars, and street food among adults; school came about among adolescents (Table 4).

**Table 4:** Energy contribution from places where food is consumed away from home, by age group. Brazil, 2017–2018.

Location	Total % (95%CI)	Adolescent % (95%CI)	Adult % (95%CI)	Older adult % (95%CI)
School	5.8 (5.2–6.3)	27.6 (24.7–30.5)	0.9 (0.7–1.1)	1.9 (0.9–2.9)
Restaurant	15.3 (14.0–16.7)	6.6 (4.4–8.7)	16.7 (15.0–18.2)	24.3 (19.1–29.5)
bar and street	12.4 (11.1–13.5)	10.6 (8.8–12.4)	13.1 (11.7–14.5)	8.0 (6.2–9.9)
Other	66.5 (64.9–68.3)	55.2 (52.0–58.5)	69.3 (67.3–71.3)	65.8 (60.3–71.2)

## DISCUSSION

The second *Inquérito Nacional de Alimentação* (National Food Survey), carried out in the 2017–2018 POF, allowed to describe, for the first time in Brazil, the evolution of an important eating habit of the Brazilian population: the out-of-home food consumption. In a ten-year period, the energy contribution of this type of consumption dropped by 3.6 percentage points.

Analyzing data from Brazilian population-based surveys carried out in 2005, 2011, and 2015, Barbosa et al.^13^ mention the economic crisis of 2015 as a cause to the reduction in out-of-home consumption among Brazilians. According to the historical series of the *Pesquisa Nacional por Amostra de Domicílios Contínua* (PNAD - Continuous National Household Sample Survey), since 2012 the unemployment rate has increased, reaching the highest point (13.7%) in early 2017. This rate remained high, with variations between 11.8% and 13.1% throughout the period of the 2017–2018 POF ^14^. Moreover, food prices did not vary significantly^15^ during this period, confirming the decrease in the Brazilian population income. These findings converge with the slight changes observed in the proportion of total expenditure on out-of-home food consumption, which rose from 31.1% in 2008–2009 to 32.8% in 2017–2018^7^.

This reduction in out-of-home food consumption may also reflect changes in the way people eat or have access to food. In the POF, the category “out-of-home food” is based on the place of consumption and the entrance of food in the household stock. Since the main objective of the POF is to assess the composition of expenditures by Brazilian families, food that comes into the household, regardless of its source, is considered to be available at home and, therefore, food within the home. Thus, ready-to-eat foods from restaurants, fast food or other establishments, if consumed at home, are classified as eating indoors.

According to the 2017–2018 POF, the relative share of ready-to-eat meals in total calories, determined by household food purchases, almost doubled between 2008 and 2018^10^. These findings follow the increasing trend in the foodservice market, growing over 200% in food prepared outside the home between 2008 and 2018, as well as the share of ready-to-eat products in the purchase of food by metropolitan households in Brazil^16^.

This consumer behavior highlights the possibility that the ingestion of ready-to-eat products occurs in a similar way inside and outside the home. There is a possibility, therefore, that the identified reduction in the frequency and energy contribution of eating away from home is a reflection of the place where the food is consumed, instead of the greater consumption of food prepared at home.

The role of excessive intake of calories through the consumption of foods prepared outside the home, including those delivered at home, has already been previously demonstrated^17^. In general, ready-to-eat meals are energy-dense, nutrient-poor^18^ and associated with excessive weight gain^19^when compared to meals prepared and consumed at home^4^.

Observing the time of consumption, it can be seen that the period normally reserved for lunch concentrated the highest percentage of food consumed away home. This may also indicate that the habit of consuming food prepared outside the home results from the work routine, which prevents the return home for meals between work shifts.

Unlike the rest of the country, in the Midwest region the frequency of out-of-home food consumption increased. The capitals of this region, in particular Campo Grande, in Mato Grosso do Sul, have one of the highest income averages compared to other capitals, which would justify this one-off increase. It is noteworthy that the value of the average monthly family expenditure on food away from home in the Center-West region was the highest in the country, equivalent to 38% of total expenditure on^7^.

The stability of out-of-home food consumption in rural areas of the country has reduced the disparity with urban areas observed in the 2008–2009 survey^9^. The purchase of ready-to-eat food away from home continues to be higher among individuals in the highest fifth of income (6 times greater than among those in the lowest fifth of income)^20^. However, the reduction of this type of consumption among individuals with higher family income (above 2 minimum wages per capita) contributed to the approximation between the extreme ranges of per capita family income.

The foods that most contributed to eating away from home (alcoholic drinks, fried and baked snacks, soft drinks, pizzas, sweets and sandwiches) continue to be ultra-processed, with high energy density, rich in free sugars, saturated fat, and low in micronutrients and fibers^21,22^. The cost of out-of-home food consumption may play a role in these choices because fried and baked snacks and sweets are cheaper when compared to traditional meals consumed away from home^23^.

The consumption of ultra-processed foods is associated with overweight, obesity, cancer, cardiometabolic risk, cardiovascular disease, and mortality from all causes^24,25^. 2019 data from the *Sistema de Vigilância de Fatores de Risco e Proteção para Doenças Crônicas por Inquérito Telefônico* (Vigitel - Surveillance System for Risk and Protection Factors for Chronic Diseases by Telephone Survey) indicate that the prevalence of overweight and obesity among Brazilian adults has increased, reaching 55% for overweight and 20% for obesity^26^. In this context, food consumption away from home must be understood as a risk factor.

There are also positive aspects to eating out. The evolution in the frequency of consumption and in the energy contribution of eating away from home among older adults may represent a greater socialization of this group, bringing them closer to the behavior of younger adults. A qualitative study carried out with informal and in-depth interviews applied in older adults living alone in Rio de Janeiro (RJ) pointed out that eating away from home promotes different types of interaction and social cohesion. The authors found that the retirement, widowhood, and the children leaving home were related to changes in the group’s eating habits and that there was a search for environments that were more conducive to new social relationships, including the habit of eating outside^27^.

It is important to note that the changes in the method of food consumption data collection (from food records in 2008–2009 to 24-hour recalls in 2017–2018) do not affect the classification of the place of food consumption, which was the same in both inquiries. A limitation that may have underestimated out-of-home consumption arises from the definition of “out-of-home food”, which does not include food prepared outside home and consumed indoors. The habit of ordering ready-to-eat food may have increased between the two surveys.

Data collection considering food consumption and place of consumption (inside or outside the home) was carried out in a representative sample of the Brazilian population only in the last two POFs (2008–2009 e 2017–2018). Based on these data, this article sought to understand how out-of-home food consumption has evolved according to demographic and socioeconomic characteristics.

The results suggest that the food groups with the highest frequency of out-of-home consumption are composed of ultra-processed items, notwithstanding the decrease in the contribution of out-of-home eating to total energy intake. In addition to the economic crisis in the country, we raised the hypothesis that the consumption of food prepared outside the home (through delivery services, for example) may explain the reduction in the frequency of individuals who reported consumption outdoors. The findings reinforce that the search for strategies to improve the diet of Brazilians must consider the source of food and the form of access the food.
