# Supplementary material for: Evolution of out-of-home food consumption in Brazil in 2008–2009 and 2017–2018
Source: Rev Saude Publica. 2021 Nov 12;55(Suppl 1):6s. doi: 10.11606/s1518-8787.2021055003221 (PMC8627152; doi:10.11606/s1518-8787.2021055003221)
Supplement: Translation

# Translation: Evolução do consumo de alimentos fora do domicílio no Brasil de 2008–2009 a 2017–2018

**Authors:** Ilana Nogueira Bezerra, Thaís M Vasconcelos, Jessica Brito Cavalcante, Edna Massae Yokoo, Rosângela A Pereira, Rosely Sichieri

**Affiliations:** I Brasil Universidade Estadual do Ceará. Centro de Ciências da Saúde. Programa de Pós-Graduação em Nutrição e Saúde e Programa de Pós-Graduação em Saúde Coletiva. Fortaleza, CE, Brasil; II Brasil Universidade Estadual do Ceará. Programa de Pós-Graduação em Saúde Coletiva. Fortaleza, CE, Brasil; III Brasil Universidade Estadual do Ceará. Programa de Pós-Graduação em Nutrição e Saúde. Fortaleza, CE, Brasil; IV Brasil Universidade Federal Fluminense. Departamento de Epidemiologia e Bioestatística. Niterói, RJ, Brasil; V Brasil Universidade Federal do Rio de Janeiro. Departamento de Nutrição Social e Aplicada. Rio de Janeiro, RJ, Brasil; VI Brasil Universidade do Estado do Rio de Janeiro. Instituto de Medicina Social. Departamento de Epidemiologia. Rio de Janeiro, RJ, Brasil

**Keywords:** Comportamento Alimentar, Serviços de Alimentação, Alimentação Coletiva, Restaurantes, Alimentos de Rua, Inquéritos sobre Dietas

## Abstract

**OBJETIVO:**

Descrever a evolução do consumo de alimentos fora do domicílio no Brasil no período 2008–2018.

**MÉTODOS:**

Foram usados dados dos Inquéritos Nacionais de Alimentação (INA) de 2008–2009 e 2017–2018, realizados com 34.003 e 46.164 indivíduos, respectivamente, para estimar a frequência de consumo de alimentos fora de casa e a contribuição desse consumo para alimentos específicos. O consumo de alimentos foi investigado por meio de registros alimentares no INA 2008–2009 e de recordatórios 24 horas em 2017–2018. As estimativas foram geradas para o Brasil como um todo, para áreas urbana e rural, para faixas de idade (adolescente, adulto, idoso) e para faixas de renda.

**RESULTADOS:**

A frequência de consumo fora de casa diminuiu 8,8% entre os dois inquéritos, sem alteração na área rural, nas regiões Nordeste e Sul e nas menores faixas de renda. Houve leve aumento entre idosos e na região Centro-Oeste. De modo geral, a contribuição do consumo alimentar fora de casa para a ingestão diária de energia também diminuiu (16,3% *vs.* 12,7%), com exceção da área rural, onde houve redução da diferença em relação à área urbana entre os dois inquéritos. O consumo de alimentos fora de casa foi reduzido para a maioria dos itens avaliados. Os alimentos mais consumidos fora de casa em ambos os inquéritos foram as bebidas alcoólicas, salgadinhos fritos e assados, refrigerantes, pizzas, doces e sanduíches.

**CONCLUSÃO:**

Em 10 anos, a prevalência de consumo de alimentos e o percentual de contribuição dos alimentos consumidos fora de casa diminuíram no Brasil, mas os alimentos ultraprocessados ainda figuram como o grupo de alimento mais consumido fora de casa.

## INTRODUÇÃO

No Brasil, poucos estudos avaliaram o consumo alimentar fora do domicílio^1^. Como mostram estudos de outros países^4^, o consumo fora de casa está associado a menor qualidade nutricional e maior ingestão de energia, carboidratos, proteínas, gorduras e sódio. Essa associação entre o hábito de comer alimentos preparados fora de casa e a pior qualidade da dieta traz preocupação, uma vez que os gastos com esse consumo vêm aumentando nos últimos anos^7^.

Usando dados de disponibilidade domiciliar de alimentos das Pesquisas de Orçamentos Familiares (POF), Claro et al.^8^ descreveram a evolução dos gastos com alimentação fora do domicílio no Brasil no período de 2002–2003 a 2008–2009. Os pesquisadores encontraram tendência de aumento nesse hábito, mais acentuada nos maiores níveis de renda, mas presente em todas as regiões do país e em áreas urbanas e rurais^8^. Entretanto, a evolução do que é efetivamente consumido fora de casa ainda não foi analisada. Assim, o presente artigo descreve, pela primeira vez, a evolução no consumo de alimentos fora de casa em 10 anos, com base nos dados dos Inquéritos Nacionais de Alimentação (INA) conduzidos junto com a POF em 2008–2009 e 2017–2018.

## MÉTODOS

Foram analisados dados provenientes do INA, módulo de consumo alimentar da POF, conduzida pelo Instituto Brasileiro de Geografia e Estatística (IBGE) em dois períodos: 2008–2009 e 2017–2018.

A partir de um Sistema Integrado de Pesquisas Domiciliares, as duas pesquisas adotaram um plano amostral por conglomerado em dois estágios, correspondente a uma “amostra mestra”, comum a todas as pesquisas domiciliares do IBGE. A amostra mestra é comporta por setores censitários, que são as unidades primárias de amostragem.

Os setores passaram por esquema de estratificação que permite gerar estimativas para as cinco regiões brasileiras, para as áreas rural e urbana e para distintos níveis socioeconômicos. Os setores foram selecionados por amostragem com probabilidade proporcional ao número de domicílios existentes no setor, dentro de cada estrato final. As subamostras de unidades primárias para as POF foram selecionadas por amostragem aleatória simples em cada estrato. As unidades secundárias foram os domicílios particulares permanentes, selecionados por amostragem aleatória simples de cada uma das unidades primárias selecionadas e estratificadas. A cada três domicílios da POF, um era selecionado para participar do INA. Na POF 2008–2009, 13.569 domicílios participaram do INA; na POF 2017–2018, 20.112.

O INA incluiu todos os moradores acima de 10 anos de idade dos domicílios selecionados, totalizando 34.003 indivíduos em 2008–2009 e 46.164 indivíduos em 2017–2018. A descrição detalhada da amostragem das duas pesquisas está disponível nas publicações oficiais do IBGE^9,10^. A coleta de dados dos inquéritos foi realizada ao longo de 12 meses, de maneira uniforme nos estratos, garantindo representatividade nos quatro trimestres do ano.

No inquérito de 2008–2009, os dados foram coletados com a aplicação, em dias não consecutivos, de dois registros alimentares com informações sobre alimentos e bebidas consumidos, tipo de preparação, quantidade, horário e local de consumo (em casa ou fora de casa). Em 2017–2018, a coleta ocorreu por meio de recordatórios de 24 horas em dois dias não consecutivos. O indivíduo era entrevistado pessoalmente por um agente de pesquisa treinado, que o interrogava e anotava todos os alimentos e bebidas consumidos no dia anterior a cada visita. O roteiro da entrevista, embasado no método de múltiplas passagens, estruturava-se em estágios sequenciais^11^. Os dados foram processados em programa desenvolvido pelo IBGE.

Na edição de 2017–2018, além dos itens já investigados na edição anterior, houve o acréscimo de “itens de adição”, com produtos usualmente adicionados a alimentos como pães, massas, bebidas etc.: manteiga/margarina, molhos e açúcar ou adoçante. Para o presente estudo, levando-se em conta que o módulo de consumo 2008–2009 não abrangeu essas informações, e que a análise desses dados apenas na edição de 2017–2018 geraria estimativas equivocadamente elevadas, excluíram-se os itens de adição da gramatura para estimar percentuais de alimentos consumidos fora de casa, sendo considerados apenas os registros espontâneos, sem perguntas de sondagem.

Em ambos os inquéritos, ao final da visita, um agente de pesquisa treinado analisou os relatos junto com o entrevistado em busca de possíveis equívocos de preenchimento e omissões de alimentos comumente esquecidos (balas, pequenos lanches etc.). Em caso de ausência ou dúvida no registro da unidade de medida utilizada, pedia-se ao entrevistado que apresentasse o utensílio ao agente para que ele pudesse lançar no sistema a medida caseira correspondente.

O consumo de alimentos fora de casa incluiu todos os alimentos e bebidas adquiridos fora de casa e ingeridos sem passar pelo estoque domiciliar. Em 2017–2018, essa informação foi coletada de forma mais detalhada, com opções para especificar o local de consumo. Para o presente estudo, avaliamos o primeiro dia de consumo alimentar, considerando consumidores de alimentos fora do lar os indivíduos que relataram o consumo de pelo menos um item fora de casa.

Questionários com dados socioeconômicos e demográficos, contendo informações sobre idade e sexo dos moradores e renda familiar per capita, foram respondidos pela pessoa de referência do domicílio. A renda familiar per capita foi estratificada com base no salário-mínimo vigente à época das pesquisas (R$ 415,00 em 2008–2009 e R$ 954,00 em 2017–2018): até 0,5 salário-mínimo, 0,5 a 1 salário-mínimo, 1 a 2 salários-mínimos e 2 ou mais salários-mínimos. A idade foi avaliada com base em três faixas etárias: adolescentes (10 a 19 anos), adultos (20 a 59 anos) e idosos (60 anos ou mais).

Os alimentos citados nos dois inquéritos foram categorizados de acordo com as características nutricionais e de consumo dos itens em 26 grupos: arroz; feijão e outras leguminosas; verduras; legumes; tuberosas; frutas; massas; panificados; bolos e biscoitos doces; salgadinhos industrializados e biscoitos salgados; carne bovina; suíno; aves; peixe e frutos do mar; embutidos; ovos; leite e derivados; doces; molhos e óleos; bebidas alcoólicas; refrescos e sucos; refrigerantes; café e chás; pizzas; salgados fritos e assados; e sanduíches.

A quantidade consumida de alimentos em gramas ou mililitro foi estimada utilizando as tabelas de medidas referidas para os alimentos consumidos no Brasil, referentes a cada inquérito^10,12^. Em seguida, a composição nutricional foi estimada a partir da mesma tabela de composição nutricional gerada para o inquérito de 2017–2018^10^.

A contribuição da alimentação fora de casa para o consumo total de cada grupo de alimentos e para a ingestão total de energia (proporção consumida fora de casa) foi estimada por meio do método da razão das médias, segundo características sociodemográficas e econômicas (faixas etárias, sexo, regiões brasileiras, áreas urbana e rural e estratos socioeconômicos). As estimativas foram geradas separadamente para cada pesquisa, e seus intervalos de confiança de 95% foram comparados para identificar mudanças no tempo.

Avaliou-se o horário de consumo dos alimentos, considerando cinco períodos diferentes: das 7 às 10 horas, das 11 às 14 horas, das 15 às 18 horas, das 19 às 22 horas, das 23 às 6 horas da manhã. Horários intermediários que fossem registrados até 29 minutos da hora foram registrados na hora anterior e horários acima de 30 minutos foram registrados na hora posterior. Por exemplo, consumos realizados entre 14:01 e 14:29 foram registrados como 14h e consumos entre 14:30 e 14:59, como 15h.

Para o INA 2017–2018, avaliamos os locais específicos de consumo, estimando a contribuição de cada um para a ingestão de energia fora de casa. Foram considerados os seguintes locais: escola (consumos reportados em ambiente escolar, incluindo universidades), restaurantes (à la carte e por quilo), bar e rua (bares, lanchonetes, fast food, comida de rua vendida por ambulantes) e outros locais (consumo fora de casa em locais não classificados nos grupos anteriores, como posto de gasolina, farmácia, supermercados etc.).

As análises foram realizadas com o software SAS versão 9.4, considerando o peso amostral e utilizando o procedimento survey para incorporar a complexidade da amostra.

## RESULTADOS

Entre 2008 e 2018 houve redução da frequência de consumo de alimentos fora de casa no Brasil, nas regiões Norte e Sudeste, na área urbana, entre homens e mulheres, adolescentes e adultos e entre os indivíduos de maior faixa de renda. Não foram observadas alterações nas regiões Nordeste e Sul, na área rural, entre idosos e nas menores faixas de renda. A região Centro-Oeste foi a única que apresentou aumento das frequências de consumo fora de casa (Tabela 1). O aumento do consumo fora de casa também foi observado entre idosos com rendas domiciliares per capita entre 1 e 2 salários-mínimos (Figura 1).

**Tabela 1:** Frequência (e intervalo de confiança de 95%) de indivíduos que consomem alimentos fora de casa, segundo variáveis sociodemográficas. Brasil, 2008–2009 e 2017–2018.

Variáveis	2008–2009 (n = 34.003)	2017–2018 (n = 46.164)
	% (IC95%)	% (IC95%)
Brasil total	40,2 (39,1–41,3)	36,5 (35,6–37,5)
**Situação do domicílio**		
Urbano	42,8 (41,5–44,0)	38,0 (36,9–39,1)
Rural	27,3 (25,4–29,3)	27,6 (26,0–29,3)
**Região**		
Norte	42,6 (40,2– 45,0)	30,5(27,8–33,1)
Nordeste	33,5 (31,9–35,1)	34,8 (33,5–36,2)
Sudeste	43,7 (41,5–45,8)	36,1 (34,3–37,9)
Sul	40,1 (38,1–43,2)	38,5 (36,3–40,5)
vCentro-Oeste	41,9 (39,2–44,7)	47,7 (45,0–50,4)
**Sexo**		
Homem	44,4 (43,0–45,9)	40,0 (38,8–41,2)
Mulher	36,3 (35,0–37,6)	33,3 (32,2–34,4)
**Idade**		
Adolescentes	48,1 (45,9–50,2)	43,4 (41,4–45,4)
Adultos	42,6 (41,3–43,9)	39,3 (38,2–40,4)
Idosos	16,1 (13,6–18,5)	19,4 (18,0– 20,8)
**Renda domiciliar per capita**		
Até 0,5 salário-mínimo	30,1 (28,0–32,2)	27,1 (25,3–29,0)
0,5–1 salário-mínimo	35,3 (33,2–37,5)	33,3 (31,7–34,9)
1–2 salários-mínimos	40,2 (38,2–42,3)	37,3 (35,6–39,0)
≥ 2 salários-mínimos	50,1 (47,7–52,4)	44,6 (43,6–46,6)

**Figura 1:** Frequência de indivíduos que consomem alimentos fora de casa, segundo renda e idade. Brasil, 2008–2009 e 2017–2018.

Resultados similares aos de frequência foram observados na contribuição energética da alimentação fora de casa para a ingestão total de energia, com redução de 3,6 pontos percentuais (pp). A maior redução foi observada na região Norte (5,6 pp), e os percentuais não se modificaram para as regiões Nordeste e Centro-Oeste. A participação da alimentação fora de casa também não se modificou para indivíduos residentes nas áreas rurais do país e para os idosos (Tabela 2).

**Tabela 2:** Evolução da contribuição energética da alimentação fora de casa segundo variáveis sociodemográficas. Brasil, 2008–2009 e 2017–2018.

Variáveis	2008–2009	2017–2018
	% (IC95%)	% (IC95%)
Brasil total	16,3 (15,7–17,0)	12,7 (12,2–13,3)
**Área**		
Urbano	17,6 (16,9–18,3)	13,4 (12,8–14,0)
Rural	10,0 (9,1–11,0)	9,0 (8,1–9,8)
**Regiões**		
Norte	16,2 (14,8–17,6)	10,6 (9,5–11,7)
Nordeste	13,5 (12,6–14,3)	12,4 (11,6–13,1)
Sudeste	17,7 (16,5–18,9)	12,4 (11,4–13,3)
Sul	16,9 (15,5–18,5)	13,9 (12,5–15,3)
Centro-Oeste	17,7 (15,6–19,8)	16,0 (14,6–17,4)
**Sexo**		
Homem	17,8 (17,0–18,6)	13,6 (13,0–14,2)
Mulher	14,6 (13,9–15,4)	11,7 (11,1–12,3)
**Idade**		
Adolescentes	15,2 (14,2–16,2)	12,2 (11,4–13,1)
Adultos	18,5 (17,7–19,3)	14,6 (13,9–15,2)
Idosos	6,5 (5,2–7,8)	5,6 (4,9–6,1)
**Renda domiciliar per capita**		
Até 0,5 salário-mínimo	11,4 (10,4–12,5)	8,2 (7,5–8,9)
0,5–1 salário-mínimo	13,2 (12,0–14,3)	10,3 (9,6–11,1)
1–2 salários-mínimos	15,8 (14,7–17,0)	12,5 (11,7–13,4)
≥ 2 salários mínimos	21,7 (20,3–23,0)	17,8 (16,7–19,1)

O consumo de bebidas alcoólicas fora de casa contribuiu com quase 50% de todas as bebidas alcoólicas consumidas em 2017–2018. Apesar da participação da alimentação fora de casa ter se reduzido em mais de 10 pontos percentuais entre os inquéritos, esse item continua sendo o que apresenta maior contribuição para a alimentação fora de casa. Salgados fritos e assados são o segundo item de maior contribuição, seguido de refrigerantes, pizzas, doces e sanduíches em 2017–2018, e pizzas, sanduíches, refrigerantes e doces em 2008–2009.

A evolução do consumo de alimentos específicos mostra redução para a maioria dos itens avaliados, entretanto perfis diferenciados podem ser observados segundo a situação do domicílio. A contribuição da alimentação fora de casa na área urbana foi maior do que na área rural para quase todos os grupos, com exceção de molhos e óleos. E a participação do consumo fora de casa da maior parte dos grupos na área rural apresentou pouca ou nenhuma alteração comparando os dois inquéritos. Tanto para a área urbana como para a rural, a contribuição diminuiu para frutas, doces e sanduíches. De modo geral, destaca-se a redução expressiva do consumo fora de casa de sanduíches (23 pp), pizzas (17,8 pp) e salgados fritos e assados (13,1 pp). A redução da participação dos sanduíches na área urbana foi superior à da área rural (23,4 pp vs. 16,1 pp) (Tabela 3).

**Tabela 3:** Evolução da contribuição dos grupos de alimentos consumidos fora de casa no Brasil e nas áreas urbana e rural. Brasil, 2008–2009 e 2017–2018.

Alimentos	Brasil		Urbano		Rural	
	2008–2009 % (IC95%)	2017–2018 % (IC95%)	2008–2009 % (IC95%)	2017–2018 % (IC95%)	2008–2009 % (IC95%)	2017–2018 % (IC95%)
Arroz	12,8 (12,1–13,5)	10,4 (9,8–10,9)	14,1 (13,2–14,9)	11,0 (10,3–11,6)	7,2 (6,2–8,2)	7,5 (6,4–8,7)
Feijão e outras leguminosas	12,2 (11,4–12,9)	9,4 (8,8–10,0)	13,6 (12,7–14,5)	10,0 (9,3–10,6)	6,8 (5,4–7,7)	6,7 (5,6–7,8)
Verduras	18,9 (17,1–20,6)	15,1 (13,9–16,3)	20,2 (18,2–22,2)	15,7 (14,4–17,0)	8,9 (6,6–11,1)	9,6 (7,0–12,2)
Legumes	13,1 (10,8–15,4)	12,9 (11,5–14,4)	14,3 (11,8–16,9)	13,9 (12,3–15,6)	7,3 (2,7–11,9)	7,0 (4,4–9,6)
Tuberosas	16,3 (14,0–18,6)	14,5 (12,7–16,2)	18,0 (15,4–20,7)	15,5 (13,5–17,6)	8,1 (5,3–10,9)	9,0 (6,5–11,5)
Frutas	15,7 (14,4–17,0)	9,5 (8,6–10,4)	16,4 (14,9–17,9)	10,1 (9,1–11,1)	12,8 (10,5–15,2)	6,2 (4,8–7,7)
Massas	14,5 (12,7–16,2)	13,0 (11,5–14,4)	15,2 (13,0–17,2)	13,2 (11,5–14,8)	9,8 (7,3–12,3)	11,5 (8,7–14,3)
Panificados	9,3 (8,6–10,0)	8,1 (7,4–8,7)	9,4 (8,6–10,2)	8,2 (7,5–9,0)	8,6 (6,8–10,3)	6,5 (5,2–7,7)
Bolos e biscoitos doces	19,2 (17,2–21,3)	16,0 (14,4–17,5)	20,4 (17,9–22,8)	16,7 (15,0–18,4)	13,6 (10,4–16,9)	11,8 (9,5–14,0)
Salgadinhos industrializados e biscoitos salgados	20,0 (16,5–23,5)	12,9 (10,9–14,4)	21,2 (16,9–25,4)	13,7 (11,3–16,1)	14,8 (10,6–19,0)	9,1 (6,8–11,5)
Carne bovina	16,2 (14,9–17,6)	13,5 (12,5–14,5)	17,8 (16,2–19,4)	14,2 (13,1–15,4)	8,5 (6,7–10,2)	9,2 (7,5–10,9)
Suíno	17,0 (12,3–21,8)	10,7 (8,5–13,0)	20,3 (14,0–26,6)	12,5 (9,5–15,4)	9,2 (4,6–13,8)	6,1 (3,7–8,4)
Aves	17,2 (15,6–18,7)	11,2 (10,3–12,1)	18,5 (16,6–20,3)	11,8 (10,8–12,8)	9,8 (7,5–12,1)	7,9 (6,3–9,5)
Peixe e frutos do mar	10,7 (8,7–12,8)	14,1 (11,4–16,8)	12,8 (10,3–15,4)	17,7 (14,0–21,3)	7,0 (3,6–10,4)	5,2 (3,9–7,0)
Embutidos	11,7 (8,9–14,6)	9,6 (7,9–11,2)	11,8 (8,7–15,0)	10,0 (8,1–11,9)	10,8 (7,2–14,4)	6,7 (4,2–9,1)
Ovos	6,7 (5,5–7,4)	6,4 (5,2–7,5)	7,5 (6,1–8,9)	6,5 (5,3–7,8)	3,8 (2,4–5,2)	5,5 (2,3–8,8)
Leite e derivados	7,9 (6,9–8,8)	6,5 (5,6–7,4)	7,9 (6,9–8,9)	8,4 (5,4–7,3)	7,5 (5,1–10,0)	6,9 (5,0–8,8)
Doces	33,2 (30,2–36,2)	23,3 (20,7–25,9)	33,7 (30,4–37,0)	24,8 (21,9–27,8)	29,8 (23,0–36,5)	15,1 (11,6–18,7)
Molhos e óleos	8,5 (7,3–9,8)	5,7 (3,6–7,9)	8,8 (7,5–10,2)	5,5 (3,2–7,7)	6,3 (4,1–8,6)	7,2 (0,4–14,0)
Bebidas alcoólicas	60,8 (53,9–67,7)	49,6 (43,7–55,4)	61,0 (53,5–68,5)	48,6 (42,4–54,8)	59,0 (44,3–73,7)	61,9 (51,4–72,4)
Refrescos e sucos	18,5 (17,3–19,8)	14,9 (13,9–15,9)	19,3 (17,9–20,7)	15,6 (14,4–16,7)	13,3 (10,9–15,7)	10,8 (9,3–12,2)
Refrigerantes	40,0 (37,4–42,4)	30,9 (28,5–33,3)	40,2 (37,5–42,9)	30,9 (28,3–33,5)	36,4 (29,5–43,3)	30,8 (25,3–36,4)
Café e chás	9,9 (9,3–10,6)	9,9 (9,2–10,7)	11,0 (10,2–11,8)	10,8 (10,0–11,7)	5,5 (4,5–6,4)	5,9 (5,0–6,9)
Pizzas	42,5 (31,9–53,1)	24,7 (18,4–31,0)	42,0 (30,7–53,3)	24,6 (18,1–31,1)	52,8 (26,5–79,2)	27,8 (10,9–44,7)
Salgados fritos e assados	48,8 (44,3–53,3)	35,7 (32,0–39,4)	51,1 (46,3–56,1)	37,0 (33,1–40,9)	29,1 (20,3–37,8)	25,3 (17,0–33,6)
Sanduíches	41,3 (36,9–45,8)	18,3 (16,4–20,1)	41,8 (37,0–46,6)	18,4 (16,4–20,4)	32,6 (22,0–43,2)	16,5 (11,6–21,4)

Em 2008–2009, a faixa de horário que apresentou maior frequência de alimentos consumidos fora do domicílio foi entre 11 e 14 horas (45,4%). Outros 19,8% foram consumidos entre 7 e 10 horas; 20,1% entre 15 e 18 horas; 12,2% entre 19 e 22 horas; e 2,5% entre 23 e 6 horas. Em 2017–2018, as frequências mantiveram a mesma ordem, sendo 39,9% dos alimentos consumidos entre 11 e 14h; 19,7% entre 7 e 10 horas; 21% entre 15 e 18 horas; 13,8% entre 19 e 22h e 5,5% entre 23 e 6 horas.

Em 2017–2018, outros locais não especificados foram os que apresentaram maior percentual de contribuição dos alimentos consumidos fora de casa, seguidos por restaurantes e bares e comida de rua entre adultos, e escola entre adolescentes (Tabela 4).

**Tabela 4:** Contribuição energética dos locais de consumo de alimentos fora de casa, segundo faixa etária. Brasil, 2017–2018.

Local	Total % (IC95%)	Adolescente % (IC95%)	Adulto % (IC95%)	Idoso % (IC95%)
Escola	5,8 (5,2–6,3)	27,6 (24,7–30,5)	0,9 (0,7–1,1)	1,9 (0,9–2,9)
Restaurante	15,3 (14,0–16,7)	6,6 (4,4–8,7)	16,7 (15,0–18,2)	24,3 (19,1–29,5)
Bar e rua	12,4 (11,1–13,5)	10,6 (8,8–12,4)	13,1 (11,7–14,5)	8,0 (6,2–9,9)
Outro	66,5 (64,9–68,3)	55,2 (52,0–58,5)	69,3 (67,3–71,3)	65,8 (60,3–71,2)

## DISCUSSÃO

O segundo Inquérito Nacional de Alimentação, realizado com a POF 2017–2018, permitiu descrever, pela primeira vez no país, a evolução de um importante hábito alimentar da população brasileira: o consumo de alimentos fora de casa. Em um período de dez anos, a contribuição energética desse tipo de consumo caiu 3,6 pontos percentuais.

Barbosa et al.^13^, ao analisar dados de pesquisas brasileiras de base populacional realizadas em 2005, 2011 e 2015, mencionam a crise econômica de 2015 como causa da redução do consumo fora de casa entre brasileiros. Segundo a série histórica da Pesquisa Nacional por Amostra de Domicílios Contínua (Pnad), a partir de 2012 a taxa de desocupação aumentou, atingindo seu máximo (13,7%) no início de 2017. Essa taxa se manteve elevada, com variações entre 11,8% e 13,1% durante todo o período da POF 2017–2018^14^. Além disso, nesse período, o preço dos alimentos não apresentou grandes variações^15^, o que confirma a diminuição na renda por parte da população brasileira. Esses achados convergem com as discretas mudanças observadas na proporção do gasto total com consumo de alimentos fora do domicílio, que passou de 31,1% em 2008–2009 para 32,8% em 2017–2018^7^.

Essa retração do consumo fora de casa também pode ser reflexo de mudanças na forma de se alimentar ou de se ter acesso aos alimentos. Na POF, a categoria “alimentação fora de casa” é baseada no local de consumo e na passagem do alimento pelo estoque domiciliar. Uma vez que a POF tem como principal objetivo avaliar a composição dos gastos das famílias brasileiras, os alimentos que entram no domicílio, independentemente de sua fonte, são considerados como disponíveis no domicílio e, portanto, alimentação dentro de casa. Assim, alimentos prontos para consumo provenientes de restaurantes, fast food ou outros estabelecimentos, se consumidos em casa, são classificados como alimentação dentro de casa.

Segundo a POF 2017–2018, a participação relativa de refeições prontas no total de calorias, determinada pela aquisição alimentar domiciliar, quase dobrou entre 2008 e 2018^10^. Esses achados acompanham a tendência de aumento no mercado de food service, com crescimento superior a 200% na alimentação preparada fora de casa entre 2008 e 2018, assim como na participação de produtos prontos para consumo na aquisição de alimentos por domicílios metropolitanos do Brasil^16^.

Esse comportamento do consumidor destaca a possibilidade de que a ingestão de produtos prontos para consumo ocorra de forma semelhante dentro e fora do domicílio. Há a possibilidade, portanto, de que a redução identificada na frequência e na contribuição energética da alimentação fora de casa seja reflexo do local em que o alimento é consumido, mas não do maior consumo de alimentos preparados em casa.

O papel da ingestão excessiva de calorias por meio do consumo de alimentos preparados fora de casa, incluindo os entregues em domicílio, já foi demonstrado em estudos anteriores^17^. Em geral, refeições prontas para consumo são densas em energia, pobres em nutrientes^18^ e associadas a ganho excessivo de peso^19^, quando comparadas às refeições preparadas e consumidas em casa^4^.

Observando o horário de consumo, percebe-se que o período normalmente reservado ao almoço concentrou o maior percentual de alimentos consumidos fora de casa. Isso também pode indicar que o hábito de consumir alimentos preparados fora do domicílio decorre da rotina de trabalho, que impede o retorno a casa para realizar as refeições entre os turnos de trabalho.

Diferentemente do restante do país, na região Centro-Oeste a frequência de consumo de alimentos fora de casa aumentou. As capitais dessa região, em particular Campo Grande, no Mato Grosso do Sul, apresentam uma das maiores médias de renda em comparação com as demais capitais, o que justificaria esse aumento pontual. Destaca-se que o valor da despesa média mensal familiar com alimentação fora do domicílio na região Centro-Oeste foi o maior do país, equivalendo a 38% dos gastos totais com alimentação^7^.

A estabilidade do consumo de alimentos fora do domicílio nas áreas rurais do país diminuiu a disparidade em relação às áreas urbanas observada no inquérito de 2008–2009^9^. A aquisição de alimentos prontos para consumo fora do domicílio continua sendo mais elevada entre os indivíduos do maior quinto de renda (6 vezes maior do que entre os do menor quinto de renda)^20^. No entanto, a redução desse tipo de consumo entre indivíduos de maior renda familiar (acima de 2 salários-mínimos per capita) contribuiu para a aproximação entre as faixas extremas de renda familiar per capita.

Os alimentos que mais contribuíram para a alimentação fora de casa (bebidas alcoólicas, salgados fritos e assados, refrigerantes, pizzas, doces e sanduíches) continuam sendo os ultraprocessados, com elevada densidade energética, ricos em açúcares livres e gordura saturada e baixo teor de micronutrientes e fibras^21,22^. O custo dos alimentos para consumo fora de casa pode desempenhar um papel nessas escolhas, uma vez que salgados fritos e assados e doces são mais baratos quando comparados a refeições tradicionais consumidas fora de casa^23^.

O consumo de alimentos ultraprocessados está associado a sobrepeso, obesidade, câncer, risco cardiometabólico, doenças cardiovasculares e mortalidade por todas as causas^24,25^. Dados de 2019 do Sistema de Vigilância de Fatores de Risco e Proteção para Doenças Crônicas por Inquérito Telefônico (Vigitel) apontam que as prevalências de sobrepeso e obesidade entre adultos brasileiros cresceram, chegando a 55% para excesso de peso e 20% para obesidade^26^. Nesse contexto, o consumo alimentar fora de casa deve ser entendido como fator de risco.

Há também aspectos positivos em comer fora de casa. A evolução na frequência de consumo e na contribuição energética da alimentação fora de casa entre idosos pode representar maior socialização desse grupo, aproximando-os de comportamentos de adultos mais jovens. Estudo qualitativo realizado com homens idosos que moravam sozinhos no Rio de Janeiro (RJ) apontou, a partir de entrevistas informais e em profundidade, que a alimentação fora do lar promove diferentes tipos de interação e coesão social. Os autores verificaram que aposentadoria, viuvez e saída dos filhos de casa estavam relacionadas a mudanças nas práticas alimentares do grupo, e que havia uma busca por ambientes mais propícios a novas relações sociais, incluindo o hábito de comer fora de casa^27^.

É importante destacar que a mudança no método de coleta dos dados de consumo alimentar (de registros alimentares em 2008–2009 para recordatórios de 24 horas em 2017–2018) não afeta a classificação do local de consumo dos alimentos, que foi a mesma nos dois inquéritos. Uma limitação que pode ter subestimado o consumo fora do domicílio decorre da definição de “alimentação fora de casa”, que não inclui alimentos preparados fora mas consumidos dentro do domicílio. O hábito de encomendar comida pronta para consumo dentro de casa pode ter aumentado entre as duas pesquisas.

A coleta de dados sobre o consumo efetivo de alimentos com classificação do local de consumo (dentro ou fora de casa), em amostra representativa da população brasileira, foi realizada somente nas duas últimas POFs (2008–2009 e 2017–2018). Com base nesses dados, este artigo buscou compreender como o consumo de alimentos fora de casa tem evoluído segundo características demográficas e socioeconômicas.

Os resultados sugerem que, apesar da diminuição na contribuição da alimentação fora de casa para a ingestão total de energia, os grupos de alimentos com maior frequência de consumo fora de casa são compostos por itens ultraprocessados. Além da crise econômica no país, foi levantada a hipótese de que o consumo domiciliar de alimentos preparados fora (por meio serviços de delivery, por exemplo) pode explicar a redução na frequência de indivíduos que reportaram consumo fora. Os achados reforçam que a busca por estratégias para melhorar a alimentação dos brasileiros deve considerar a fonte do alimento e a forma de acesso.
